# 705. Community Acquired Gastrointestinal Infections among Transplant Recipients

**DOI:** 10.1093/ofid/ofab466.902

**Published:** 2021-12-04

**Authors:** Mohanad Al-Obaidi, Tirdad T Zangeneh

**Affiliations:** University of Arizona, Tucson, Arizona

## Abstract

**Background:**

Community-acquired gastrointestinal (cGI) infections carry a significant risk of mortality and morbidity. Transplant patients are at increased risk of infectious complications. We aimed to study the risks and outcomes of cGI infections in this population.

**Methods:**

After the institutional review board’s approval, a multi-center retrospective study was conducted. Data was collected from inpatient admission for patients with a history of hematopoietic stem transplantation or solid organ transplantation. Data regarding patient demographics, gastrointestinal polymerase chain reaction (GIPCR), clinical presentation, medications, discharge, and length of stay were collected. Chi-square test was performed to compare categorical data, and student’s t-test and Wilcoxon test were used to compare parametric and non-parametric variables accordingly.

**Results:**

From 10/01/2017 to 07/14/2020, there were 445 encounters with GIPCR tests ordered. 48% were female, 53% were non-Hispanic White, and the mean age was 58 (SD ±14.6). Of the 445 encounters, 66 had a positive test. 40/66 had kidney transplants. The most common detected organisms were Norovirus (36%), Enteropathogenic *E. coli* (26%), *Campylobacter* species (9%), and Enteroaggregative *E. coli* (9%). The most common symptoms were abdominal pain and diarrhea, with 26% reported an exposure or a recent travel. There was no difference in the mortality rates between positive and negative GIPCR (3% versus 2.4%, p=0.7), during the study period. There was a significant difference in the mean length of stay between positive GIPCR with 7.5 (SD ±10.5) days versus 12.4 (SD ±18.3) days in negative GI PCR, p=0.036.

**Conclusion:**

The majority of GIPCR tests were negative. Patients with positive GIPCR had shorter length of stay compared to negative GIPCR transplant recipients. There was no difference in mortality between positive and negative GIPCR among transplant patients. Future studies are required to evaluate the impact of cGI infections on transplant patients.

**Disclosures:**

**All Authors**: No reported disclosures

